# The Usefulness of Nanotechnology in Improving the Prognosis of Lung Cancer

**DOI:** 10.3390/biomedicines11030705

**Published:** 2023-02-24

**Authors:** Gabriela Bordeianu, Nina Filip, Andrei Cernomaz, Bogdan Veliceasa, Loredana Liliana Hurjui, Alin Constantin Pinzariu, Mihaela Pertea, Andreea Clim, Mihai Vasile Marinca, Ionela Lacramioara Serban

**Affiliations:** 1 Department of Morpho-Functional Sciences (II), Discipline of Biochemistry, Faculty of Medicine, “Grigore T. Popa” University of Medicine and Pharmacy, 700115 Iasi, Romania; 2 III-rd Medical Department, Discipline of Pneumology, “Grigore T. Popa” University of Medicine and Pharmacy, 700115 Iasi, Romania; 3 Department of Orthopedics and Traumatology, Surgical Science (II), Faculty of Medicine, “Grigore T. Popa” University of Medicine and Pharmacy, 700115 Iasi, Romania; 4 Department of Morpho-Functional Sciences (II), Discipline of Physiology, Faculty of Medicine, “Grigore T. Popa” University of Medicine and Pharmacy, 700115 Iasi, Romania; 5 Department of Plastic Surgery and Reconstructive Microsurgery, “Sf. Spiridon” Emergency County Hospital, 700111 Iasi, Romania; 6 III-rd Medical Department, Discipline of Oncology, “Grigore T. Popa” University of Medicine and Pharmacy, 700115 Iasi, Romania

**Keywords:** nanomedicine, lung cancer, drugs, nanoparticles

## Abstract

Lung cancer remains a major public health problem both in terms of incidence and specific mortality despite recent developments in terms of prevention, such as smoking reduction policies and clinical management advances. Better lung cancer prognosis could be achieved by early and accurate diagnosis and improved therapeutic interventions. Nanotechnology is a dynamic and fast-developing field; various medical applications have been developed and deployed, and more exist as proofs of concepts or experimental models. We aim to summarize current knowledge relevant to the use of nanotechnology in lung cancer management. Starting from the chemical structure-based classification of nanoparticles, we identify and review various practical implementations roughly organized as diagnostic or therapeutic in scope, ranging from innovative contrast agents to targeted drug carriers. Available data are presented starting with standards of practice and moving to highly experimental methods and proofs of concept; particularities, advantages, limits and future directions are explored, focusing on the potential impact on lung cancer clinical prognosis.

## 1. Introduction

The birth of the term nanotechnology is anecdotally linked to the American physicist Richard Feynman in the early 1960s; currently, this is an umbrella term for technologies dealing with structures between 1 and 100 nanometers [1]. Nanotechnology has established a foothold in the medical space; it is employed in various branches spanning from diagnosis to treatment. Atheroma plaque healing, regenerating damaged nerves, and targeting tumor tissues are only a few examples of the practical applications of nanotechnology [2].

Recent years have brought significant improvements concerning survival and quality of life for various hematological and solid malignant disease patients; lung cancer lags behind despite new emerging therapies [3]. Various strategies have been devised to improve lung cancer outcomes; early diagnosis and better therapeutic options are focus points. Early diagnosis enables radical therapeutic procedures such as surgical resection or curative intent radiotherapy while maintaining quality of life; however, in the case of lung cancer, late onset of clinical signs, lack of reliable biomarkers, and imaging-related limitations make such an approach difficult [4].

Lung cancer therapeutic protocols are usually chosen considering the histology of the tumor and the extension and mainly combine surgery, radiation therapy, and chemotherapy. For non-resectable lung cancer, the prognosis is linked to oncological treatment efficacy, which is generally limited by tolerability and toxic side effects [5]; immuno- and targeted therapy are recent additions gaining ground and leading towards personalized medicine [6] but are adequate only in a minority of cases.

Nanotechnologies could alleviate some conventional therapy drawbacks and improve efficacy; tailoring drug pharmacokinetics (by facilitating intra- and intercellular traffic or navigating the tumor micro-environment), targeting various cellular lines, and modulating the immune response are only a few possibilities [7]. 

Nanoparticles is an umbrella term encompassing a plethora of chemically different structures potentially useful in both early diagnosis and better therapy.

We review the available nanotechnologies and their potential role in lung cancer management, underlining advantages, weak points, and particularities.

## 2. Nanoparticles

Targeting points of interest is a thorny problem in general pharmacology, and this is particularly so in oncology, where specific drugs often have less than ideal biodistribution, with a reduced effect on tumor cells and toxic consequences on normal structures.

Some of these distribution problems may be tackled by using nanoparticles (NPs)—chemical structures able to contain drugs and direct them to various targets. Their nanostructures have physicochemical characteristics conferring them biocompatibility and making them adequate for the task [8,9]. NPs may be roughly classified based on their nature as polymeric, lipid-based, or inorganic. Each class presents advantages and disadvantages in terms of size, physicochemical properties, geometry, as well as bioavailability [7].

### 2.1. Lipid-Based Nanoparticles

The most well-known, FDA-approved and used class of NPs is lipid-based; these particles include liposomes and lipid nanoparticles. Liposomes have a vesicular structure and consist of phospholipids that provide a uni- or multi-lamellar structure with an aqueous environment inside. This structure allows the incorporation and transport of hydrophilic, hydrophobic, or lipophilic drugs [10,11].

Liposomes may be modified. For example, adding peptides may confer better tissue penetrability even through biological barriers and allow new routes of drug delivery [12]. Polyethylene glycol (HO–(CH_2_CH_2_O) n–H) is soluble in both water and non-polar organic solvents and may be conjugated with various nanoparticles. PEGylated liposomes show improved circulation time and better tumor accumulation [13,14]; however, the presence of PEG-specific antibodies may limit the usefulness of such an add-on as anaphylaxis or complement-mediated destruction of the liposome may occur [15,16,17].

Solid lipid nanoparticles have a micellar structure with outfacing cationic head groups and a lipophilic interior. Their composition is made up of lipids with melting points higher than body temperature [18,19]. Such structures are generally stable and versatile and may be used to contain and protect drug molecules; their lipid structure may be altered to fine-tune their properties [20,21]. Solid lipid nanoparticles are adequate for nucleic acid encapsulation and delivery [22]; the first approved application was a nanoparticle-embedded interference RNA structure (Onpattro) used to treat polyneuropathies. Other developments are deemed possible, such as embedding promoters/enhancers for some genes useful in cancer therapy [8,21].

### 2.2. Polymeric Nanoparticles

Polymeric nanoparticles may be roughly classified as nanocapsules, which are lipophilic cores surrounded by a polymeric membrane-like structure, and nanospheres, which are solid polymeric network structures. They are mainly used as delivery systems where the payload is carried in the core of the nanocapsules or absorbed into the nanospheres [23,24,25]. Polymeric nanoparticles may also be classified as polymersomes, micelles, and dendrimers [7].

Polymersomes are vesicle-like structures bearing similarity to liposomes but made up of amphiphilic polymers [26]; they contain an aqueous medium that can be used to encapsulate hydrophilic molecules, including chemotherapeutic agents, enzymes, proteins, and nucleic acid structures [27]. Their chemical and biological properties, such as membrane robustness or stability, may be modified by using different copolymers. PEG may be added to the surface as a sheath with the effect of sterically limiting rejection and improving the plasma half-life [28]. 

The copolymers used to build NPs may be responsive to stimuli such as pH variations, temperature, ionic concentrations, and magnetic fields. This allows for the controlled release of the payload; targeting may be further improved as various guiding components may be added to polymeric surfaces, including antibodies, antigenic structures, and peptides [29]. 

Micelles bear similarities to liposomes excepting the internal aqueous space. They are self-assembled structures with a diameter in the range of 5–50 nm made up of amphiphilic agents (either lipidic or polymeric) with their polar sites facing outwards [26]. Generally, micelles are readily uptaken by tumor cells, which makes them suitable as drug carriers; for example, there are data on docetaxel-conjugated micelles used as oral therapy for squamous cell carcinoma resulting in improved pharmacokinetics [30]. Similarly, there are data suggesting micelle encapsulation may provide a solution for bypassing biological barriers and delivering the payload in particular conditions, such as in the case of doxorubicine-loaded micelles crossing the encephalic barrier and having a biological effect on otherwise resistant glioma cells [31]. 

Dendrimers are three-dimensional NPs with a diameter in the range of 2–10 nm [32]; they usually have an arborescent structure centered on a core from which repetitive units irradiate. The external layer of the dendrimer presents functional groups; the number and nature of those may be altered, and thus, the chemical and biological properties of the dendrimer may be changed [33]. 

### 2.3. Inorganic NPs

Inorganic NPs are a heterogeneous group. Metal-based NPs (gold, silver, iron), oxides (iron oxide), semiconductor structures (quantum dots), carbon dots and nanotubes are among the best studied [34], though there are many sizes, shapes, and structures available. There are data connecting at least some inorganic NPs to undesirable biological reactions, such as promoting inflammation, fibrosis, or tumorigenesis (the link between lung cancer and carbon nanostructure exposure is documented) [35,36]. 

Still, inorganic NPs have physical properties that may recommend their use in both diagnostic and therapeutic technologies. The majority of FDA-approved inorganic NPs are iron-oxide-based (Fe_3_O_4_—magnetite or maghemite) as these compounds are biocompatible and nontoxic; their magnetic properties make them particularly suited as contrast agents. Some therapeutic developments have also been reported, such as in the magnetic induced hyperthermia of tumor tissue [37].

Similarly, gold NPs have photothermal properties exhibiting a localized surface plasmon resonance phenomenon. They also accumulate in tumor tissue and can be targeted using a plethora of conjugates and thus might be used in therapy [38,39,40].

Other common inorganic structures with potential therapeutic applications include calcium phosphate and mesoporous silica NPs, which have both been used successfully for gene and drug delivery [7,41]. 

While developing new therapies is paramount, early detection remains an extremely important survival predictor, particularly for lung cancer patients. The last decades have brought new imaging techniques, but lung cancer is still a problem as its diagnosis is often established in advanced stages. 

### 2.4. Quantum Dots 

Quantum dots are semiconductor nanocrystals with a diameter in the 2–10 nm range mainly used as fluorescent dyes [34,42]. They consist of a metallic core (Cd, Pb, Zn, Ga, or In) coated with a shell (usually ZnS) and a cap that improves solubility [43,44]. Their in vivo lifetime is longer, and their signal strength/concentration ratio is better than fluorescent dyes. Such properties make quantum dots potentially useful for in vivo examinations such as fluorescence bronchoscopy to detect in situ carcinomas, and they may also have a role in photodynamic therapies [45,46]. We summarized the advantages and disadvantages of the main types of nanoparticles from a pragmatic point of view in Table 1.

Although there are encouraging data concerning the role of NPs in the diagnosis and therapy of some conditions (including neoplastic disease), their exact place is still not clear. Much data come from in vitro and animal model research, and long-term effects have not been thoroughly assessed. Some NPs are already included in mainstream medical applications, and relevant safety data will probably accumulate. Still, their heterogenous nature and the possible combinations available make a full pharmacological characterization of NP practically impossible.

## 3. Nanotechnologies and Lung Cancer Diagnosis

Establishing a clear positive lung cancer diagnosis may be a complex and tedious enterprise—clinical, biological, imagistic and histopathology data must be collected and interpreted in a process that is difficult to standardize.

Early lung cancer stages are associated with better therapy response, longer survival, and even complete healing. Stage I cases have a 5-year survival rate of almost 80%, although this drops to less than 15% for stage III and IV patients [71].

Clinical elements (signs and symptoms) are either non-specific for lung cancer or develop slowly, thus leading to late presentation. Histology (or less effective cytology) examinations are usually required to formulate the lung cancer diagnosis and to properly manage such a case. Obtaining such samples requires some form of imaging data [72].

Imaging is probably the cornerstone of lung cancer diagnosis. Thoracic computed tomography is the most important component, but standard chest X-rays, various ultrasound examinations, magnetic resonance imaging, and other methods also play a role. Any abnormal result is followed by tissue sampling techniques (bronchoscopy, guided needle biopsy, open surgery) if a neoplastic nature is deemed probable [73]. Lung cancer diagnosis is inextricably linked to tissue sampling, but this process is always triggered and guided by imaging, from chest X-rays to computed tomography and PET-CT. Thus, improving the sensitivity and specificity of these methods will have an indirect impact on survival. 

An early cancer diagnosis may be systematically sought by implementing population screening policies and procedures; lung cancer screening is currently limited to serial low-dose CT examinations. Recent data showed that lung cancer screening may be effective (albeit expensive) for selected population subgroups, but this approach is still plagued by false positives and negatives, unnecessary interventions, and high radiation exposure. There is consensus about the need to improve existing methods and develop new ones to improve the early detection of lung lesions [4,74]. 

Making better use of existing biomarkers (lowering prices and time to results and increasing availability), developing/implementing new ones, and improving imaging techniques are logical directions for lung cancer screening development and are fields for nanotechnology deployment.

### 3.1. Imagistics

Iodine compounds have been historically linked to classic radiology examinations, and they are still the main contrast agents associated with computed tomography. These molecules are generally small and have a fast rate of renal clearance. To improve their pharmacodynamics, various nanoparticle carriers have been considered, such as MPEG-iodolysine copolymers and various iodine-based emulsions (such as iodinated triglycerides) [75,76,77,78]; increasing circulating lifetime might reduce the necessary contrast dose [79]. 

Iodine-free contrast solutions have also been considered, such as metal nanoparticles. Gold was deemed a good candidate due to its optical properties, biocompatibility, and ability to attach to targeted surface structures [56]; nanoparticle affinity for tumor cells may allow the development of functional and tumor-targeted imaging methods. 

Among the imaging technologies, magnetic resonance has the evident advantage of not involving ionizing radiation. In magnetic resonance imaging, images are generated from signals originating in nuclear spin variation following magnetic field changes and reflect the water content of the examined structures [80]. 

The imaging process is time-intensive, and thus, movement interference is important. However, low pulmonary water content and respiratory and cardiac cycles limits its uses for lung lesions. Still, magnetic resonance imaging (MRI) has its uses in lung cancer patients as it offers detailed information on thoracic blood vessels, mediastinal structures, and various metastasis-prone extrathoracic sites [81]. MRI contrast is typically gadolinium-based. Nanoparticles may improve pharmacokinetics and thus decrease the dose and associated toxicity. Semiconductor polymers, porous silica particles, and nanodiamond conjugates have been tested and showed increased signal strength while decreasing the administered dose of elemental gadolinium equivalent [82].

Superparamagnetic iron oxide nanoparticles, dubbed SPIONs, were tested as a gadolinium-free contrast alternative, showing promising results in terms of biocompatibility and signal boosting. One advantage was the hepatic elimination route, which allows their use in renal failure patients [83].

Along the line of gadolinium-free MRI contrast, ferumoxytol, initially approved as iron replacement therapy for renal failure patients, may be useful. Ferumoxytol is taken up by the macrophages and transported to the reticuloendothelial system, thus being particularly useful for lymph node imaging [84].

High equipment and tracer costs and inflammatory lesion uptake limit the usefulness of positron emission tomography–computed tomography (PET-CT) scanning as a screening tool; it is mainly used for disease staging once the diagnosis has been confirmed with superior results to standard CT [85,86]. 

64 Cu and 177 Lu encapsulated into PEGylated liposomes showed improved tumor uptake with a better tumor-to-muscle ratio, thus facilitating data interpretation. Furthermore, liposomes may be targeted by adding guidance molecules such as somatostatin for neuroendocrine tumors [87,88].

### 3.2. Biomarkers

Biomarkers play a significant role in the early diagnosis of some cancers; such is the case of prostate-specific antigen (PSA) for prostate cancers or CA19–9 for solid digestive tumors. In fact, some screening strategies are built around such tests. Currently, there is no single biomarker accepted for lung cancer early detection, although some are spuriously used, such as neuron-specific enolase (NSE) for small cell lung cancer or cytokeratin for squamous lung cancer. The need for different biomarkers for various histology types makes the use of such tests difficult and expensive. Nanotechnology may partially address these problems. Available non-laboratory-based electrochemical biosensor arrays may be coupled with detector particles (peptides, aptamers) to develop a specific diagnostic tool [89].

Aptamers are stable and reusable and may be coupled to multiple signaling structures (fluorophores, enzymes, other nanosystems), making them particularly useful in detecting promising novel lung cancer biomarkers, such as circulating DNA or micro-RNA [89]. High levels of circulating RNA were reported for various types of NSCLC and seem to correlate with tumor burden and clinical outcome. Cost and availability may be a problem that may be circumvented by using a quantum-dot-based nano biosensor [90,91]. 

Furthermore, there are quantum-dot-based detection element multiplex solutions able to assess multiple markers at once (NSA, Cyfra 21-1, and carcinoembryonic antigen (CEA)) with lower detection thresholds compared to classic biochemistry methods [92,93]. 

Similarly, there are developments in chip-based microfluidic-based systems able to detect and analyze free tumor cells using patient blood samples. Such an approach may be useful to assess the driver mutation status (such as epidermal growth factor receptor (EGFR)) without the need for a tissue sample, thus improving the outcome of the current liquid biopsy approaches [94,95]. 

A summary of current nanoparticle imagistic applications relevant to lung cancer diagnosis is presented in Table 2.

## 4. Nanotechnologies and Lung Cancer Therapy

Lung cancer remains one of the most frequently diagnosed malignant diseases; despite some progress in prevention, early detection, and advanced therapy, its prognosis is usually severe, and associated mortality remains high. Tobacco smoking was identified as the main risk factor, and some population-level risk mitigation measures have been implemented; other external factors, such as air pollution (environmental and domestic), also have a role [5]. Surgery, radiotherapy, and oncological therapies are the main pillars of lung cancer treatment; early diagnosis and correct staging are paramount to optimize outcomes [6]. 

Classic oncological management of lung cancer involves chemotherapy, though recent molecular biology developments have brought out new therapeutic methods with increased efficacy and better safety profiles, such as targeted agents and immunotherapy. 

Paclitaxel is a chemotherapic agent frequently used in breast, ovary, prostate, and lung cancer protocols. The doublet paclitaxel platinum salt may be considered the mainstay of non-small cell lung cancer therapy. Paclitaxel acts as a tubulin-binding agent, stopping mitosis and promoting cellular death; it has low hydro-solubility, and therefore, an emulsifier vehicle is necessary, usually the solvent oil cremophor ethanol (CrEL). The administration of a CrEL-paclitaxel formula may be followed by potentially lethal adverse events such as hypersensitivity reactions, peripheral neuropathy, or myelosuppression [103,104,105]. Various mitigation strategies are used in clinical settings, such as corticoid and antihistamine premedication and low infusion rates; one potential alternative may be the use of albumin-bound paclitaxel nanoparticles, which seem to have increased plasma life and antitumor activity, at least in murine human tumor xenograft models [106]. The nab-paclitaxel formula (under the trade name Abraxane) was initially FDA-approved in 2004 for metastatic breast cancer; in 2012, it was accepted for the first-line treatment of locally advanced or metastatic non-small cell lung cancer in combination with carboplatin in patients who are not candidates for curative surgery or radiation therapy [107]. Using the nab-paclitaxel form allowed enhanced tumor penetration and cellular uptake [108] (probably by transporter-mediated mechanisms) with a higher clinical response rate and a better safety profile than classic CrEL paclitaxel [109]. There are data supporting the use of nab-paclitaxel as higher effective concentrations can be reached with a shorter infusion time, eliminating the need for premedication used to alleviate the risk of solvent-induced hypersensitivity reactions [110].

Another way to improve the chemical stability and solubility of CrEL-paclitaxel made use of liposomes; the cytotoxic effect was similar to classic Taxol, but bioavailability and stability were improved. In 2006, a formulation was approved in China under the trade name LIPUSU [86]. There are data suggesting that higher cellular uptake and better cytotoxicity with a similar safety profile may be attained by altering the lipid components of the liposomes (by adding lysophosphatidylcoline by a simple process) [111].

Other paclitaxel nanoparticles are under scrutiny. Polymeric micellar paclitaxel (pm-Pac) is a CrEL-free structure that was recently tested in phase III trials, showing increased tumor cell penetration and reduced adverse effects in combination with cisplatin, thus potentially becoming a new chemotherapy option for advanced non-small-cell lung cancer patients [112,113]. Polylactic-co-glycolic acid (PLGA) has also been considered as a potential paclitaxel carrier; there are published data supporting higher cytotoxicity, stronger apoptosis signal, weaker migration and invasion for NSCLC cells when using solvent-based paclitaxel as a comparator [114,115].

Doxorubicin is a potentially useful chemotherapeutic agent for many solid tumors [116,117], but its high toxicity and induced resistance may impose limits on its use. Particularly for lung cancers, doxorubicin shows low cellular penetration, low tumor concentrations, and significant toxicity [118,119]. Various nanoparticle–doxorubicin delivery systems have been tested, and some showed better pharmacokinetics and bioavailability and a lower effect on normal cells [95,114].

The dimercaptosuccinic acid terminated poly (amido-amine) (PAMAM) dendrimers conjugated with doxorubicin proved effective in delivering doxorubicin using a glucose moiety as a targeting structure and making use of the increased glucose uptake of tumor cells; furthermore, the dimensions of the conjugates decreased the renal elimination and demonstrated a longer half-life [120]. 

Doxorubicin-containing PEGylated liposomes are available and widely used in clinical oncology (PLD; CAELYX, Schering-Plough Corp., Kenilworth, NJ, USA/DOXIL, ALZA, Mountain View, CA, USA) as toxic effects (mainly cardiotoxicity and myelosuppression but also vomiting and alopecia) are mitigated compared with conventional doxorubicin [114,121,122].

Molecular cancer targets are products of so-called driver mutations. The most frequent are EGFR, KRAS (Kirsten rat sarcoma virus gene), HER2 (human epidermal growth factor receptor 2 gene), ALK (anaplastic lymphoma kinase), ROS1 (tyrosine-protein kinase ROS gene), cMET (MNNG HOS transforming gene), BRAF (B-Raf gene), RET (rearranged during transfection gene), and NTRK (neurotrophic tyrosine receptor kinase gene) [123].

The best-known driver mutations for NSCLC involve the epidermal growth factor receptor gene (EGFR), being detected in 10–15% of lung adenocarcinoma patients. EGFR is a receptor tyrosine kinase, a member of the ErbB family, and may be physiologically activated by multiple ligands; this interaction may activate various intracellular signaling pathways, such as PI3K/AKT/mTOR, Ras/Raf/MEK/ERK1/2, and the phospholipase C (PLC) cascade—with clear implications regarding cell migration, attachment, angiogenesis, and organogenesis regulation [123,124].

Various activating EGFR mutations have been documented; the in-frame exon 19 deletion and the L858R substitution account for 85% of relevant driver mutations in 85% of NSCLC cases, but there are multiple deletions, insertions, point mutations and duplications reported concerning exons 18–25 [125].

Such mutations may lead to persistent signal pathway activation with decreased apoptosis and cell proliferation and play a role in tumorigenesis; therefore, the EGFR domains became a potential target for novel antitumor agents [126].

The first generation of EGFR tyrosine kinase inhibitors (EGFR-TKI) has a reversible effect on the tyrosine kinase EGFR domain. Various clinical trials have shown improved survival for mutation-harboring patients using standard cytotoxic therapy as a comparator [127,128]. Although the survival rate has improved, the patients acquire resistance to these drugs after 9–14 months [129,130].

EGFR exon 20 T790M deletion, which occurs in 50–60% of NSCLC patients undergoing first-generation EGFR-TKI therapy (such as erlotinib or gefitinib), is the most common mechanism of acquired resistance; the second generation of EGFR-TKI (afatinib and dacomitinib) was developed aiming to circumvent this drawback without noticeable success [131].

The 3rd-generation EGFR-TKI (osimertinib, rocelitinib, olmutinib) proved to be effective in overcoming the resistance induced by the T790M deletion and are currently considered first-line agents in NSCLC protocols for patients with driver mutations [132].

Similar to classic antitumor agents, the idea of boosting the effects of EGFR-TKIs using nanoparticles was investigated. There are data concerning the use of GEF-loaded poly(ε-caprolactone)-poly(ethyleneglycol)-poly(ε-caprolactone) (PCEC)-bearing nanoparticles (GEF-NPs) with improved antitumor effects, prolonged survival time, and less side effects using classic gefitinib as a comparator [133].

Human serum albumin (HAS) is non-immunogenic and has ideal biocompatibility. It is frequently used as a drug vehicle as it improves the solubility of lipophilic drugs. Hyaluronic acid (HA) is a negatively charged polysaccharide that is similarly biocompatible and known to interact with some surface molecules such as CD-44, lymphatic vessel endothelial receptor-1, and receptor for hyaluronan-mediated motility that are frequently overexpressed in malignant cells [134,135]. An erlotinib/hyaluronic acid/human serum albumin complex (ERT-HSA-HA NPs) was developed and tested on tumor cell lines and animal models with promising results, including tumor growth inhibition and lack of recurrence, possibly explained by longer plasma half-life and higher tumor uptake [136,137].

Complex associations were also tested. Doxorubicin and icotinib (proven more effective than erlotinib and apatinib) were encapsulated using cationic amphipathic starch and hyaluronic acid. The resulting NPs were tested using lung cancer lines and murine models and were shown to accumulate in tumor cells with a smaller effect on normal cells [10].

Both afatinib and dacomitinib (a second-generation, irreversible EGFR-TKI, FDA-approved) have low solubility, which translates to low pulmonary tissue bioavailability; such a drawback might be circumvented by a direct administration route by using a system of poly-(lactic-co-glycolic-acid) nanoparticles (PLGA NPs) developed for inhalation for pulmonary lesions [138].

Osimertinib is the first FDA-approved third-generation EGFR-TKI; current therapeutic protocols allow its use for both NSCLC patients with activating EGFR mutation and patients with T790M resistance mutation cancers with encouraging results, though still limited by acquired resistance. Among the strategies laid out to overcome osimertinib resistance, the use of complex nanoparticles might play a role; a combination of osimertinib and selumetinib (a MEK inhibitor with limited NSCLC effects) conjugated with PEG using a reactive oxygen species-responsive linker had encouraging in vitro and murine model effects. The PEG-selumetinib complex acted as a micelle carrier for osimertinib and delivered the drug payload in high reactive oxygen species activity zones such as tumor cells; such an approach may combine the benefits of both targeting tumor cells and preventing acquired resistance [139].

The association of nanotechnologies and EGFR-TKIs is not limited to lung cancer therapy. A creative combination of erlotinib and superparamagnetic iron oxide core particles was found to exhibit affinity towards EGFR overexpressing cells. Such an approach may enable MRI-based detection of EGFR mutated tumors; this would be valuable as MRI techniques are generally of little use for lung imaging despite some obvious advantages, such as no ionizing radiation exposure [140].

The anaplastic lymphoma kinase (ALK) gene is located on chromosome 2 and en-codes a transmembrane tyrosine kinase that normally has a low expression in small intestine, nervous system, and testicular cells in adults. Still, ALK gene rearrangement was reported in some NSCLCs; its prevalence is between 3% to 7% in adenocarcinoma cases; many current diagnostic protocols include routine ALK testing for relevant histology samples [141,142]. 

The c-ros oncogene 1 (ROS1) codes a tyrosine receptor kinase belonging to the insulin receptor family; some rearrangements have been reported particularly in adenocarcinoma cases occurring in young, never smoking patients (Asian descent may also play a role). Multiple ROS1 mutations have been reported (with various signaling pathways involved). Their global prevalence is estimated between 1 and 3% of lung adenocarcinomas [143].

Crizotinib is a tyrosine kinase inhibitor active on ALK, MET and ROS1 available in oral form; its effectivity is limited by various mechanisms such as mutations in the ALK kinase domain, the increased number of ALK fusion genes, and central nervous system progression stemming from low penetration of the blood–brain barrier [144,145].

Polymeric nanoparticles based on polylactide-tocopheryl polyethylene glycol 1000 succinates (PLA-TPGS) may be used to encapsulate crizotinib with better cellular uptake and increased biological effect [146].

Similarly, poly (ethylene glycol)–poly(ε-caprolactones)–poly (ethylene glycol) (PEG–PCL–PEG, PECE) structures have been used as delivery systems for both sorafenib and crizotinib (SORA-CRIZ-NPs), improving their hydrosolubility and reducing their toxic effects [147]. 

Alectinib was approved in 2015 and is included in current therapeutic protocols for ALK-positive NSCLC cases with resistance or progression under crizotinib therapy [148,149]. Various side effects such as anemia, increased aminotransferase activity, hyperbilirubinemia, and hyperglycemia affect most users [150]. 

One modern oncological approach to lung cancer is the relatively new check-point immunotherapy; this method makes use of immunoglobulins to prevent the interaction between the programmed death ligand-1 (PD-L1) and its receptor (cluster of differentiation 274 (CD274) or PD-1) the underlying mechanism being T-cell cytotoxic mediated [151,152,153]. Silencing PD-L1 and PD-1 on tumor-infiltrating lymphocytes by deploying siRNA on a lipid-coated calcium phosphate carrier proved to be an effective approach in a breast cancer model, suggesting a way to improve immunotherapy outcomes [153].

There are some completed clinical trials (Table 3) investigating various nanotechnology therapeutic applications in the field of lung cancer. There is considerable variability in terms of investigative products and efficacy endpoints; we have compiled a list using the keywords lung cancer and nanoparticles on clinicaltrials.gov site (accessed on 20 December 2022).

This makes it difficult not only to identify the best therapeutic options but also to identify which way further research should focus. Despite one phase IV trial, the majority of completed trials are phases I and II, which may imply additional data are still required to validate the use of some nanotechnologies in clinical practice.

Current guidelines hold chemotherapy, mutation-targeted therapy, and immunotherapy as standard approaches in lung cancer management, along with surgery and radiotherapy. Nanoparticles may play a role as adjuvants to radiotherapy and topic minimal invasive interventions such as photodynamic therapy. A summary of potential applications is presented in Table 4.

**Figure 1:**
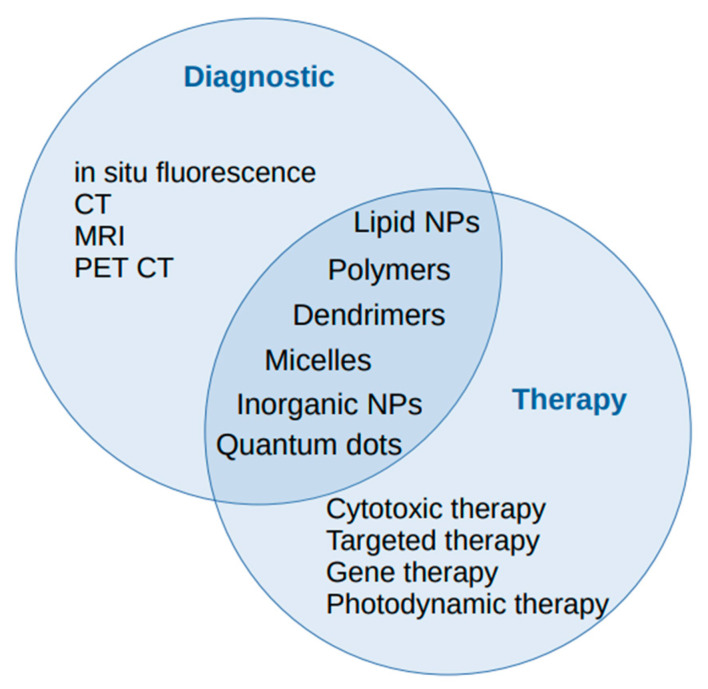
The diagnostic and therapeutic roles of nanoparticles in lung cancer management.

From a practical point of view some nanoparticles exhibit peculiar properties enabling potential multiple roles at once, both diagnostic and therapeutic (Figure 1). This represents an emerging concept dubbed theranostics. Such an approach is the use of quantum dots as a fluorescent agent able to guide and amplify the biological effect of bronchoscopy-delivered photodynamic therapy, an intervention particularly suited to carcinoma in situ management [158,169]. Similarly, near-infrared emitting QDs were experimentally successfully used to improve the intra-operatory visualization of pulmonary nodules and establish resection limits. The resulting in vivo fluorescence proved to be relatively independent of dimensions and vascularization and allowed improved detection beyond CT data [169,170].

## 5. Limits and Drawbacks

Although theoretical, proof of concept, and model data are encouraging regarding nanoparticle-driven therapeutic interventions, there are few data originating in stage III and IV clinical trials, and those data that exist are mainly concerning lipid particles used as carriers for either standard chemotherapeutics or tyrosine kinase inhibitors [3]. Furthermore, most of the available data were obtained from advanced disease groups where the therapeutic effects are generally minimal. There are few data, and most are highly experimental and relevant to nanoparticle-based diagnosis of lung cancer.

Nanoparticles, as an umbrella term, covers multiple structures with different chemical properties, and there are limited data available concerning their safety profile. There are data pertaining to acute or chronic toxicity following nanoparticle exposure; the effects are difficult to assess as the mechanisms involved may vary: direct cytotoxic effects, oxidative stress, or secondary inflammation have been hypothesized [171,172]. Establishing a nanoparticle safety profile is further complicated by organ deposition and accumulation [173,174]. There are abundant data concerning deleterious lung effects such as fibrosis and cancerogenesis, albeit for inhaled nanoparticles [175,176].

Encouraging data originating from animal experiments and cell lines should be carefully extrapolated as tumor models are limited in scope when compared with real-life scenarios and do not always scale well. Systemic reactions such as immune reactions or local factors such as tumor stroma microenvironment or cellular heterogeneity may alter the hypothesized effects [8,177,178]. Along the same line, in vivo particle stability may differ from in vitro experimental data. Mechanisms such as unspecific protein adsorption have been reported [179].

Some nanoparticles have stability issues, which may complicate their storage and shorten their shelf life or further increase costs [180].

## 6. Conclusions

Nanotechnologies are increasingly used in lung cancer management; there are potential applications ranging from clinical suspicion through the diagnosis process to treatment options. Some nanoparticles have been investigated and adopted in standard clinical protocols, mainly lipid-based particles used as drug carriers. Various developments and strategies are currently under scrutiny, such as new nanoparticles exhibiting microenvironment-targeting properties or adding active targeting components such as peptides or immunoglobulins, though limited experimental data on these are available. Imaging is central to lung cancer diagnosis, and nanotechnologies may be used to improve the sensitivity of existing protocols, enable the use of non-standard methods such as magnetic resonance for lung lesions, or open new directions in functional imaging. The advent of applied medical nanotechnology development has created new terms such as ‘theragnostics’: the use of metallic nanoparticles as contrast agents and simultaneously as radiotherapy sensitizers is a good illustration of this concept. Cost and difficulties in obtaining clinical trial data are potential obstacles to advancing nanotechnology applications in lung cancer management. The creation of multidisciplinary teams is critical to lead new developments.

## Figures and Tables

**Table 1:** Main types of nanoparticles with medical applications.

Nanoparticles Type		Advantages	Disadvantages	Reference
Liposomes		- Able to encapsulate hydrophilic and hydrophobic drugs - Allow the targeting of specific sites by adding proteins to the membrane (some of these structures may be stimulus-sensitive structures) - Their stability can be altered by changing the composition of the lipid membrane (e.g., adding cholesterol) - Can carry and protect DNA - Stability and hydrophilicity can be improved by PEGylation	- High production cost - Low solubility - Short half-life - Fusion or premature release of encapsulated molecules	[47,48,49,50,51,52]
Solid lipid nanoparticles		- Low cost - Easy to produce - Generally nontoxic - Site-specific targeting - Chemical stability in solutions and protection of labile drugs	- Reduced drug transport capacity - The alteration of the structure in time (e.g., polymerization) - Accumulation in liver and spleen (some)	[19,53,54,55]
Polymeric nanoparticles	Natural polymers	- Biocompatibility - Biodegradable - Less toxicity - Prolong blood circulation times of encapsulated drugs	- Expensive to produce - Variability in material from animal sources - Complexity of structure	[56,57,58,59]
	Synthetic polymers	- Biocompatibility	- Expensive to produce - Toxic - Non-biodegradable	[56,60,61]
Metallic nanoparticles		- Variable pharmacokinetics depending on the dimensions - Allow magnetic targeting	- Low biocompatibility and toxicity due to contaminants - Tendency for hepatic and splenic accumulation	[62,63,64,65,66]
Quantum dots		- Compact structure - Alternative fluorescent probe (can emit light spectrum from visible to infrared) - Photostable, narrow emission spectra	- Their components (cadmium) can be toxic to human cells	[67,68,69,70]

**Table 2:** Nanoparticle applications for lung cancer diagnosis.

Procedure	Nanoparticles	Role	Reference
Fluorescence—in situ examination	Fluorescent and non-fluorescent NPs (e.g., quantum dots, silica-coated with fluorophores)	Fluorescent agents	[34,42,96]
Computer tomography	Gold nanoparticles	Targeted contrast agent	[79,97]
Magnetic resonance imaging	SPIONs, gadolinium oxide-based NPs, manganese oxide NPs	Improved contrast agents	[83,98,99,100]
Positron emission tomography	Gd_2_O_3_-doped carbon-11-choline (GdCho), gold/mesoporous silica hybrid nanoparticles, manganese oxide NPs	Improved contrast agents	[99,101,102]

**Table 3:** Completed clinical trials indexed on clinicaltrials.gov relevant to nanoparticle-augmented lung cancer therapy.

Study Type	Description	Primary Outcome	NCT Number	Number of Participants
Phase IV	Efficacy and safety of paclitaxel liposome and cisplatin compared with gemcitabine and cisplatin as first-line therapy in advanced squamous non-small-cell lung cancer	Progression-free survival	NCT02996214	536
Phase II	ABI-009, human albumin-bound rapamycin, in patients with metastatic, unresectable, low, or intermediate grade neuroendocrine tumors of the lung or gastro-enteropancreatic system who have progressed or been intolerant to everolimus	Disease control rate	NCT03670030	5
Phase II	Safety and efficacy of BIND-014 (docetaxel nanoparticles for injectable suspension) as second-line therapy to patients with non-small-cell lung cancer	Objective response rate	NCT01792479	64
Phase II	BIND-014 (docetaxel nanoparticles for injectable suspension) as second-line therapy for patients with KRAS positive or squamous cell non-small cell lung cancer	Disease control rate	NCT02283320	69
Phase II	Carboplatin and paclitaxel albumin-stabilized nanoparticle formulation together with radiation therapy and erlotinib in treating patients with Stage III NSCLC that cannot be removed by surgery	Overall survival at 12 months	NCT00553462	78
Phase II	Paclitaxel albumin-stabilized nanoparticle formulation given together with carboplatin in treating patients with stage IIIB, stage IV, or recurrent NSCLC	Overall response rate	NCT00729612	63
Phase I-II	Side effects and optimal dose of ABI-007 (paclitaxel albumin-stabilized nanoparticle formulation) efficacy in treating patients with stage IV NSCLC	Target lesion response (safety, tolerability, antitumor activity)	NCT00077246	64
Phase II	CRLX101 (camptothecin (CPT) conjugated to a cyclodextrin-based polymer) vs. best supportive care (BSC) in advanced non-small-cell lung cancer (NSCLC)	Overall survival	NCT01380769	157
Phase II	Paclitaxel albumin-stabilized nanoparticle formulation (Abraxane) in treating patients with previously treated advanced non-small-cell lung cancer.	Overall response rate	NCT01620190	26
Phase I/II	Safety and antitumor activity of ABI-007 (a unique protein formulation of paclitaxel) in weekly administration in naïve patients with advanced non-small cell lung cancer	Establishing the toxicity	NCT00073723	75
Phase I	TargomiRs (targeted minicells containing a microRNA mimic) as 2nd or 3rd line treatment for patients with recurrent malignant pleural mesothelioma and non-small-cell lung cancer.	Establishing maximum tolerated dose and dose-limiting toxicities	NCT02369198	27
Phase II	Effectiveness of nab-paclitaxel + carboplatin + MPDL3280A (monoclonal antibody directed against the protein ligand programmed cell death-1 ligand 1 (PD-L1) for treatment of non-small-cell lung carcinoma (NSCLC)	Major pathologic response rate	NCT02716038	39
Phase I/II	Combination therapy with NC-6004 (nanoparticle-cisplatin) and gemcitabine in patients with advanced solid tumors or non-small-cell lung, biliary, and bladder cancer	Progression-free survival	NCT02240238	209

**Table 4:** Nanoparticle-enabled lung cancer therapeutic procedures.

Procedure	Nanoparticles	Role	Reference
Photothermal therapy	Gold nanoparticles, Fe_3_O_4,_ polydopamine	Fluorescent dye, photosensitizer, theragnostic agent	[154,155,156,157]
Photodynamic therapy	Quantum dots, photosensitizer nanoparticles (hypocrellin B)	Photosensitizer	[158,159,160,161]
Radiation therapy	Gold and platinum-based NPs	Sensitizer	[162,163,164]
Gene therapy	Liposomal nucleic acid delivery system (lipofectamine), solid lipid- and polymer-based gene delivery vectors	Nucleic acid delivery systems	[165,166,167]
Chemotherapy	Polymers, dendrimers, liposome-based drug delivery systems (various chemotherapeutic agents)	Carriers, targeted carriers	[112,115,120,121,168]

## Data Availability

Not applicable.
